# ‘Corona? 5G? or both?’: the dynamics of COVID-19/5G conspiracy theories on Facebook

**DOI:** 10.1177/1329878X20946113

**Published:** 2020-11

**Authors:** Axel Bruns, Stephen Harrington, Edward Hurcombe

**Affiliations:** Queensland University of Technology, Australia

**Keywords:** 5G, conspiracy theory, coronavirus, COVID-19, disinformation, dissemination, Facebook, misinformation, rumours, social media

## Abstract

Focussing in detail on one key component of the infodemic surrounding COVID-19, this article traces the dissemination dynamics of rumours that the pandemic outbreak was somehow related to the rollout of 5G mobile telephony technology in Wuhan and around the world. Drawing on a mixture of quantitative and qualitative methods including time-series analysis, network analysis and in-depth close reading, our analysis shows the dissemination of the rumour on Facebook from its obscure origins in pre-existing conspiracist groups through greater uptake in more diverse communities to substantial amplification by celebrities, sports stars and media outlets. The in-depth tracing of COVID-related mis- and disinformation across social networks offers important new insights into the dynamics of online information dissemination and points to opportunities to slow and stop the spread of false information, or at least to combat it more directly with accurate counterinformation.

## Introduction

The novel coronavirus pandemic that has spread from its origins in Wuhan, China, around the globe since late 2019 has been accompanied by a similarly global ‘infodemic’, as the World Health Organization (WHO) has pointed out (Ghebreyesus, in United Nations, 2020b). This is unsurprising: the outbreak of the mystery disease now generally labelled COVID-19 and its gradual spread through the population necessarily resulted in many uncertainties about the severity and mode of transmission of the virus, about effective protection mechanisms and remedies, about government initiatives to detect the virus and slow its spread and about the longer-term personal, social and economic impacts of the social distancing, quarantine and lockdown measures put in place. At times of significant uncertainty, the circulation of rumours, misinformation and outright falsehoods tends to increase substantially, however (Allport and Postman, 1946): while official news and government information remains sketchy or contradictory, citizens are more likely to supplement their news diet with unverified information from less reliable sources, and may favour information that aligns with their pre-existing worldviews, downplays the severity of the crisis, or presents convenient external scapegoats that can be blamed for the disruption to their lives.

In modern times, such infodemics are aided by the ease with which misinformation – ‘misleading information created or disseminated without manipulative or malicious intent’ (UNESCO, 2018: 7) – from alternative sources can be accessed and shared, especially online. This is also exacerbated by the activities of individuals, groups and organisations that deliberately aim to create confusion and discord through disinformation – ‘deliberate (often orchestrated) attempts to confuse or manipulate’ (UNESCO, 2018: 7), for economic or political reasons, or simply to disrupt public communication processes. The COVID-19 crisis provides a wealth of examples for such infodemic dissemination processes – and much as the tracing of actual viral contagion provides new insights into the dynamics of pandemics and highlights the critical inflection points at which such contagion might be slowed or stopped, the in-depth tracing of COVID-related mis- and disinformation across social networks offers important new insights into the dynamics of online information dissemination and points to opportunities to slow and stop the spread of false information, or at least to combat it more directly with accurate counterinformation.

Focussing in detail on one key component of the infodemic surrounding COVID-19, this article traces the dissemination dynamics of rumours that the pandemic outbreak was somehow related to the rollout of 5G mobile telephony technology in Wuhan and around the world. The rumour also builds upon a series of related narratives regarding the possible health and environmental impacts of 5G technology, and arguably flourished in large part thanks to the pre-existing networks and misinformation surrounding it. These rumours fit very strongly into the category of ‘conspiracy theory’, which itself arguably forms a separate subset of the mis- and disinformation categories noted above. On the one hand, it is likely that conspiracies are promulgated by people who actually believe them (and want to let others ‘in’ on their knowledge), so this is presumably done with good intentions. On the other hand, conspiracy theories can be leveraged and amplified by malicious actors to stoke general discord, discredit an opponent or critic (such as when, in May 2020 via Twitter, Donald Trump indirectly accused TV host Joe Scarborough of once murdering an intern; see Baker and Astor, 2020) or to distract attention away from malfeasance or incompetence (the previous example might apply here, too). Finally, conspiracy theories sometimes (although extremely rarely) fall into neither category, because they reference something that does have a partial or complete basis in reality. To be absolutely clear, though, the connection between COVID-19 and 5G that is at the centre of the conspiracy theories we examine here is entirely fictional.

The 5G connection is not the only COVID-19-related conspiracy, of course (one of the more prominent theories, for example, claims that the virus was developed in a laboratory in Wuhan, from where it was accidentally or intentionally released). Such theories circulate perhaps because people have been seeking some more tangible (rather than literally invisible) causes for such massive social and economic disruptions.^1^

Yablokov (2015) argues that conspiracy theories ‘function by helping to unite the audience as “the people” against the imagined “Other”, represented as a secretive “power bloc”’ (p. 302). It is no surprise, then, that so many have popped up that pivot around perceived intrusions upon freedom and civil liberties by governments, billionaires, the media or medical experts. One of the most significant – and ‘mainstream’ – of these is the idea that climate change is an invention by climate scientists, designed either to help secure more government funding for their research or – in more nefarious versions – to lay that groundwork that justifies a single world government. The rich vein of scepticism regarding the safety and benefits of vaccines work similarly, and are founded on a suspicion of modern medicine (with those ascribing to this belief often seeking out alternative, ‘natural’ remedies), and towards science and technology more broadly.

Although it is hard to provide a complete picture of the interrelationship between the two ideas, it is notable that one of the main ‘hot spots’ for anti-5G protests in Australia – Mullumbimby in Northern New South Wales (NSW) (see Bibby, 2020) – is in an area which has traditionally had some of the lowest child vaccination rates in the country (MacKenzie, 2017). So, while we study this case in isolation, it shares dynamics with long-standing popular conspiracy theories (Amarasingam, 2019), albeit with new, pressing public health concerns in the context of a pandemic (Argentino, 2020). The gradual spread of the idea from fringe conspiracist groups to celebrity endorsements, mainstream media coverage and official government and WHO denials (e.g. Australian Government, 2020; UK Government, 2020; United Nations, 2020a) also demonstrates what are likely to be typical dissemination processes for such rumours.

We focus on the COVID/5G rumour for the purpose of this article because, of the various COVID-related misinformation stories, it has arguably generated the most immediate and most visible impacts – in early April 2020, several mobile phone towers in the United Kingdom, the Netherlands, and other countries, as well as some of the technicians servicing them, were attacked by believers in the rumour (Osborne, 2020). This demonstrates that such mis- and disinformation does not necessarily remain limited to online circulation, but can result in substantial offline harm. We focus in this article on the dissemination of the rumour on Facebook as the most widely used global social media platform; subsequent work will supplement this study with analyses of similar processes on Twitter and coverage in mainstream and fringe online news media outlets. Drawing on a mixture of quantitative and qualitative methods including time-series analysis, network analysis and in-depth close reading, our analysis shows the dynamics of the rumour from its obscure origins in pre-existing conspiracist groups through greater uptake in more diverse communities to substantial amplification by celebrities, sports stars and media outlets.

## Methods and dataset

We draw for our analysis on data accessed through Facebook’s metrics platform CrowdTangle, which covers public pages, public groups and verified profiles on the platform only. This limitation is entirely appropriate as it protects the privacy of Facebook users, but we note that this means that we are necessarily unable to trace the further dissemination of COVID/5G rumours through closed groups, private or semi-private personal profiles (e.g. profiles whose posts are available only to friends or friends of friends) or direct messaging; our analysis here is limited to the fully public spaces on the wider Facebook platform. For the purposes of this study, however, this is appropriate and sufficient: we are interested here predominantly in the major flows of rumour transmission, which are likely to run most swiftly in widely visible public spaces, rather than in the more minor flows that may branch off from this stream.

CrowdTangle is predominantly designed for commercial social media analytics users. Previously a third-party audience analytics tool, it was acquired by Facebook in 2016 (Newton, 2016) and is used mainly by Facebook’s commercial partners. More recently, CrowdTangle has been made available increasingly to leading scholarly social media research centres and institutes around the world. At this stage, although there is an open application form for prospective academic users, scholarly access to CrowdTangle is still provided mainly by invitation. We acknowledge that this creates divisions within the Internet research community, and – in line with our previous arguments on this point (Bruns, 2019) – continue to call for greater scholarly access to social media data. It is also important to note in this context that academic CrowdTangle access is provided free of any limitations relating to the agenda, methodology or subject matter of research activities.

We searched CrowdTangle using the query *(covid,corona,virus,epidemi,pandemi) AND (5g)*; this identified any posts on public groups, pages and verified profiles that contained any of the five words or word stems related to the COVID-19 outbreak *together with* the term ‘5G’, which is universally used to refer to this technology. We employed ‘epidemi’ and ‘pandemi’ as word stems in this search as they might thus capture ‘epidemic’, ‘epidemiologists’ and other related terms. Furthermore, the use of these terms also produced results from a broad range of the languages used on Facebook: many of these terms (especially ‘COVID’ and ‘corona’) are used in identical form across languages, and often even in languages that otherwise employ non-Latin scripts. However, we note that our data coverage is nonetheless more likely to be comprehensive for languages that employ Latin script than it is for others. More generally, of course, the global coverage of our dataset is shaped by the uptake of Facebook as a communication platform around the world: in particular, as Facebook is generally inaccessible in mainland China itself, and plays a secondary role to VKontakte in Russia and some other post-Soviet republics, the dissemination of COVID-related rumours in these countries is likely to occur predominantly on other social media platforms.

We focus our analysis in this article on the period from 1 January to 12 April 2020; this timeframe covers the gradual spread of information about the pandemic around the world, the implementation of increasingly severe quarantine and lockdown measures in most nations and the attacks on mobile phone towers in the United Kingdom and elsewhere in early April. This does not mean that rumours relating to the connections between COVID-19 and 5G technology have stopped to circulate, of course – indeed, recent public statements by the WHO and national governments that sought to correct and debunk such rumours may in fact have served to embolden the conspiracists promoting such rumours, as they can now point to such statements as further evidence of a global conspiracy to cover up ‘the truth’. But here we are interested predominantly in the early phases of such rumour dissemination, which had clearly concluded by the time that the rumours prompted such physical attacks on mobile telephony infrastructure.

In total, then, the CrowdTangle dataset produced by our search query contains some 89,664 distinct Facebook posts on public pages, public groups or verified profiles. In the analysis that follows, we first retrace the dynamics of the rumour dissemination by combining computational time-series analysis with a close reading of key posts in order to illustrate the gradual spread of rumours from the fringes to more popular spaces in the Facebook platform. We pay particular attention also to the types and reach of these spaces, to highlight the ways in which such spaces would have amplified these rumours to new communities. Finally, we also examine networks of URL sharing in order to identify the broader communities of pages, groups and profiles that promoted similar COVID/5G content and examine what common attributes they share.

In order to avoid a further overt amplification of these rumours and conspiracy theories, in what follows we generally only summarise them and refrain from including direct quotations or screenshots from Facebook posts or the outside resources they may link to; we believe that extensive quoting from the source material could increase the platform for harmful conspiracy theories and extend their circulation, and that publication of such content in a scholarly journal could enhance the persistence of messages that would otherwise disappear or be explicitly removed from Facebook itself. We do explicitly identify a number of sources that come to play a particularly pivotal role in the dissemination dynamics for the COVID/5G rumours, however.

Furthermore, in order to assess the likely spread, visibility and thus impact that particular posts achieved, we focus here especially on the number of followers that the public pages, groups and verified profiles had at the time of posting. Although of course following a page does not mean that a user would see every post from that page, and we cannot therefore determine the *absolute* number of Facebook users who would have seen one post or another, this metric is nonetheless a useful *relative* measure of the virality of any given post, in comparison with other posts in our dataset. Both through views on these pages themselves and through further private or semi-private on-sharing, a post appearing mainly in pages and groups with a few hundred followers would be far less likely to reach a large audience than a post that appeared in pages and groups with millions or tens of millions of followers.

We must also note here, however, that CrowdTangle’s reporting of the ‘likes at posting’ metric for pages, groups and profiles is somewhat incomplete: it captures this number only for the substantial list of pages, groups and profiles it is tracking on an ongoing basis and provides no value for pages, groups and profiles that appear only because their posts matched our search terms but are not tracked beyond this. Our data will thus systematically underestimate the total number of followers for the pages, groups and profiles that shared a given post, and this is an inevitable aspect of working with CrowdTangle data on Facebook communication patterns – but again, our purpose in using this metric is not to produce an absolute estimate of the number of users who would have seen a post, but to provide a relative measure of different posts’ viral dissemination.

Finally, for the sake of simplicity we will refer to the public pages, public groups and verified profiles that are covered in our dataset collectively as Facebook *spaces* in the following analysis and discussion. This is warranted because – although *in general* pages, groups and profiles do play different roles in Facebook’s overall communicative ecosystem – *public* pages, groups and profiles are all accessible to any user of the platform even if they have not chosen to ‘like’ or ‘follow’ them, and their content will show up in searches conducted by Facebook users. For the purposes of our analysis, therefore, their function as public sources of information, opinion and rumour on the platform is equivalent.

## Analysis

**Figure 1.**
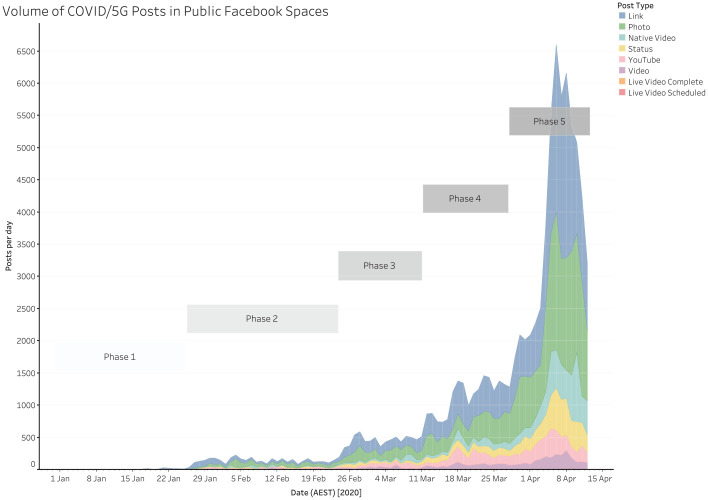
Volume of COVID/5G posts on Facebook over time, split into Facebook post types.

As Figure 1 shows, the volume of Facebook posts, in public spaces, that match our search terms increases gradually over the period we analyse here – slowly at first and then more and more rapidly. Indeed, the spread of such posts across Facebook can be loosely split into a number of distinct phases.

### Phase 1: pre-existing conspiracy theories (1–26 January 2020)

During the first weeks of January, very few Facebook posts match our selection criteria; indeed, even those that do relate only very loosely, if at all, to the COVID-19 outbreak. Rather, these posts repeat typical conspiracist tropes about secret plans to exterminate the majority of the world’s population through a combination of water fluoridation, mass vaccination, chemtrails, genetically modified foods and ‘toxic’ 5G emissions (that is, they are mostly a continuation of the extant anti-science/technology rhetoric). It remains unclear at this stage whether this claim is related directly to early reports about the COVID-19 outbreak or represents a more general, pre-existing fear. (The term COVID-19 itself was introduced by the WHO only on 11 February 2020, and these early posts are therefore necessarily somewhat vague about their subject; cf. WHO, 2020.)

Some such posts explicitly re-share much older Facebook posts that anticipate that the active extermination phase will be triggered by a ‘pandemic virus’, however, and this act of re-sharing can be interpreted as making a deliberate link between such pre-existing conspiracy theories and the present outbreak. In such sharing, we see early signs of the incorporation of current events into existing conspiracist worldviews; such ideologically coloured readings of news events are likely to be common for most conspiracist groups and indeed, in a less hyperpartisan form, are a fundamental feature of any media consumption process (cf. Hall, 1980).

The link is made explicit in an article from the French-language blog *Les Moutons Enragés* (*The Rabid Sheep*), posted on 20 January and circulating across 13 French Facebook spaces with anti-5G, anti-vaccination, alternative health and *Gilets Jaunes* protest movement sympathies that collectively had at least 65,000 followers at the time. Here, the author discusses the virus outbreak, notes an existing fringe theory that 5G emissions have negative health impacts and claims that Wuhan had begun a substantial rollout of 5G technology. According to our dataset, this represents the first time that the claim of a Wuhan-5G connection circulated publicly on Facebook.

### Phase 2: the Wuhan-5G connection spreads globally (27 January to 24 February 2020)

The *Moutons Enragés* article fails to break through the language barrier, however, and circulates only for a few days. Another article on the same topic is more successful and is posted across 43 spaces with a collective number of at least 665,000 followers: this piece, posted on 27 January on the German-language alternative medicine site *connectiv.events*, claims that Wuhan is located in the Chinese province that first rolled out 5G telephony networks and links to an open letter from ‘180 scientists and physicians’ that warns of the dangers of 5G technology (including ‘flu-like symptoms’). It adds substantial further material claiming to provide evidence for these dangers and calls for better regulation from the European Union, as well as asking for donations to support the running of its site.

Somewhat slower to circulate but eventually reaching an even larger audience of 87 spaces with a combined total of at least 1.1 million followers is a similar article that was posted, also on 27 January, in a discussion forum on the entertainment site *allkpop*. Subsequently deleted from the forum, the surviving article headline ‘Wuhan was the province where 5G was rolled out, now the center of deadly virus’ provides a clear indication of its overall theme. This article constitutes the first English-language post outlining the COVID/5G claims that circulated widely on Facebook; while it is no longer possible to determine its original contents, it may well also have drawn on the claims made in the earlier French and German posts. Although written in English, it is notable that the article link was shared widely across many language communities on Facebook, from Sweden to Pakistan and from Spain to Hong Kong.

This post is, in turn, also referenced in an article on the conspiracist blog *Vigiliae.org*, posted the following day. Here, however, the story changes somewhat: in a rambling collection of excerpts from a wide variety of sources, the article claims that the Wuhan National Biosafety Laboratory was engaged in experiments with dangerous pathogens, that the coronavirus could therefore be a medical experiment or bioweapon, and that 5G might play a role in its activation. Of these early articles drawing connections between COVID-19 and 5G, this article circulated most widely, reaching 127 spaces with at least 1.3 million followers at the time of posting; it continued to circulate even in early April, towards the end of the period we examine in this article. The article was further reposted in the US-based fringe news site *The Liberty Beacon*, from where it was shared across a further 53 spaces with at least 490,000 followers. Spaces sharing these articles again represented a wide range of locations, languages and interests, especially including the various strands of conspiracy theories it combined.

It is notable that these articles emerge around the time of a major policy decision made by the British government at the end of January, allowing the Chinese-owned telecommunications company Huawei to participate in the development of the United Kingdom’s 5G telephony network against the explicit advice of the US government (Kleinman, 2020). Indeed, as Figure 1 shows, there is a substantial increase in the total volume of Facebook posts linking COVID-19 and 5G from 27 January onwards, from fewer than 30 posts per day to between 80 and 220 posts per day during February. This is unlikely to be coincidental: at the very least, the UK decision and subsequent vocal disagreement from the US administration would have drawn considerable public attention towards 5G technology, and this may have amplified the circulation of 5G-related conspiracy content as well; the authors and sharers of COVID/5G posts may also have been drawn to this topic by such public controversy.

### Phase 3: localisation and embellishment (25 February to 11 March 2020)

While such stories continue to circulate relatively unchanged for much of February, towards the end of the month there is a notable phase shift. At this point, we see a number of new posts in languages other than English circulate widely: these include a long native Facebook post in Romanian from 26 February that is shared across 159 Romanian spaces with a total of more than 3.9 million followers, claiming that the coronavirus is only the pretext for distributing a deadly vaccine which will be activated by 5G radiation and will lead to a mass depopulation of Earth at the behest of a shadowy cabal including George Soros and Bill Gates; a video interview with a purported UK nurse discussing the alleged health effects of 5G, including a claim that 5G ‘destroys oxygen’ (posted on 29 February, shared across 141 spaces with some 1.8 million followers, and now taken down by YouTube); a lengthy post from the alternative health site *Electric Sense*, posted on 4 March, that collates various concerns about 5G technology and its health effects and suggests that 5G could make COVID-19 more virulent (127 spaces with at least 1.4 million followers); a Facebook video from 7 March that claims that viruses do not exist and that COVID-19 symptoms are instead created by 5G emissions as part of a global UN depopulation plan called ‘Agenda 21/30’ (shared across 156 spaces with at least 3.5 million followers).

It is especially notable that during this time the range of language communities addressed by such content increases substantially. In the period of 25–29 February alone, as the volume of Facebook posts addressing COVID-19 and 5G suddenly rises sharply from around 120 to between 330 and 580 posts per day, many of the most widely shared posts and links are in eastern and southern European languages including Romanian, Czech, Croatian, Italian, Spanish and French. It is impossible from the outside to determine any definitive reasons for this diversification, but it may be related to the gradual imposition of quarantine and lockdown measures in these nations; Romania, for example, announced quarantine requirements for all travellers arriving in the country on 24 February (Budu, 2020). As the COVID-19 crisis thus began to affect local populations, this may have also prompted local conspiracy theorists to engage more directly with the topic (and in doing so to draw from the English-language material already circulating through Facebook and other social media platforms); lockdowns may also have given them more *time* to do so. A more sinister explanation could be that such localised conspiracy content was also disseminated deliberately by state or other disinformation actors seeking to destabilise these countries, and the focus on eastern and southern Europe would be consistent with the past activities especially of the Kremlin-linked ‘Internet Research Agency’ troll farm in St. Petersburg (Chen, 2015) – however, at this point we do not see any definitive evidence of such deliberate activity in our data.

During this phase, as the brief outlines of some of the most prominent posts demonstrate, we also see further embellishment of the COVID/5G rumours – sometimes in diverging and contradictory directions. These are often driven by the pre-existing obsessions of the various conspiracy groups: while some continue to focus centrally on the supposed ill health effects of 5G radiation itself (even to the point of denying that there is a coronavirus at all, and claiming that COVID-19 is merely a cover story to explain the effects of the 5G tests in Wuhan), others now position 5G as part of a much larger and more complex agenda that involves bioengineered viruses and deadly, 5G-activated vaccines. In such conspiracy theories, a growing array of conspiracy bogeymen are variously said to be behind the outbreak: Bill Gates, George Soros, the WHO, the United Nations, Big Tech firms including Huawei, China and/or the United States, or secretive global conspiracy organisations such as the Illuminati.

### Phase 4: the oxygen absorption claim and lockdown suspicions (12–28 March 2020)

Another phase shift occurs from mid-March onwards, and may well be linked to the increasing implementation of lockdown measures around the world. As such lockdowns affect a growing number of people around the world, some celebrities caught up in the crisis also begin to post their own interpretations of events, sometimes echoing conspiracist talking points. On 16 March, for instance, African-American R&B singer Keri Hilson uses a series of tweets to endorse the theory that Africa had so far seen relatively few COVID-19 cases because it was not a priority region for the global 5G rollout. Screenshots of these tweets, as well as news and entertainment reports about Hilson’s views and their swift withdrawal at the behest of Hilson’s management circulated widely on Facebook in the following days, resulting in considerable circulation of these stories in the United States, Africa and Southeast Asia. The most widely shared articles about Hilson’s comments (from Nigerian newspaper *The Nation*, Kenyan news platform *Tuko*, Indonesian TV newscast *Liputan 6* and entertainment sites *Rappler, Complex* and *Popular Superstars*) were shared across 15 spaces with at least 39 million followers. Although such articles almost universally ridiculed Hilson’s views, they nonetheless also substantially amplified the content of her tweets (which most included verbatim).

Indeed, in the days following Hilson’s posts and their coverage, we also see some significant circulation of COVID/5G rumours among African communities for the first time; it is not possible for us to determine whether Hilson was herself influenced by these posts or whether her tweets encouraged them, but we note the close proximity of these occurrences: from 16 March, a lengthy Facebook post with accompanying video (both now removed) claims that 5G radiation reduces the ability of the human body to absorb oxygen, and that COVID-19 is only a cover story for the symptoms this produces. This circulates across some 275 spaces on topics ranging from religion through music to politics, with a combined total of at least 28.7 million followers. A second, shorter version of this post circulated from 17 March and reached another 153 spaces with at least 13 million followers, again mostly in Africa. The oxygen absorption claim is also at the centre of an article on the Bulgarian alternative health and spirituality site *Portal 12*, which similarly circulates from 16 March onwards. Eventually taken down from the site, it is shared across some 130 spaces with at least 6.4 million followers.

These posts appear to draw to some extent on the earlier interview with the UK nurse, who also referenced the oxygen absorption claim; more centrally, they appear to be inspired by the claims of the US-based 5G conspiracist Joe Imbriano, whose own YouTube videos about the supposed dangers of 5G had already circulated for some time without reaching a similarly large audience. One such video, shot in Imbriano’s car while driving and posted on 29 January, was shared on only three Facebook spaces with at least 200,000 followers during the timeframe studied here; a second, now removed by YouTube, appeared on 1 March and eventually reached 11 spaces with at least 194,000 followers. It is therefore only through the take-up of the oxygen absorption claim by others, especially within the African Facebook community, that this conspiracy theory is disseminated to much larger audiences.

Similarly, as lockdown measures are implemented in various US states, a YouTube video claiming that the lockdown is merely a cover to install 5G masts on school buildings across the country while teachers, parents and pupils are away begins to circulate widely; first shared on 17 March, it eventually reaches some 531 spaces and groups with more than 8 million followers. Notably, this circulation is not limited to the United States alone, however: the video is shared by spaces from Canada to Israel and from Romania to Australia. A related post on *Take Back Your Power*, a site that campaigns against smart power meters, continues this story by collating a number of videos purporting to show 5G installation vans near schools and encourages readers to contact their local schools to enquire about such activities. First seen on 22 March, it is shared by 118 spaces with more than 1.4 million followers.

From 22 March, a variety of posts in Italian also circulate widely; this is very likely prompted by the shutdown of all non-essential businesses and total domestic travel ban announced late on 21 March by Italian Prime Minister Guiseppe Conte (Safi et al., 2020). An article in the alternative health site *Oasi Sana* (*Healthy Oasis*) points to tweets by the Belgian economist Gunter Pauli, allegedly an economic advisor to the Italian Prime Minister, that repeat the claim that Wuhan was the first location in the world to roll out 5G technology and add a further claim that northern Italy, which saw the country’s worst COVID-19 outbreak, was the first European region to deploy 5G; an article in *Affari Italiani* (*Italian Affairs*), now deleted, similarly connects COVID-19, vaccines and 5G; and a lengthy Facebook video by the journalist Nicola Porro, raging against the new lockdown restrictions although he tested positive for COVID-19 himself, is shared widely especially among supporters of the far-right former Interior Minister, Matteo Salvini. Collectively, these three posts alone reach some 112 spaces with at least 1.1 million followers.

In Poland, too, an article from the conspiracist site *Odkrywamy Zakryte* (*We Uncover Secrets*), which had circulated on smaller spaces since 16 March, purporting to cover findings from biologist Paul Doyon about the supposed links between 5G and COVID-19, reached larger spaces as national lockdown measures were strengthened during the second half of March. Eventually, it is shared by nine spaces with at least 3.7 million followers.

### Phase 5: arsonists, evangelists and celebrities (29 March to mid-April 2020)

Suspicions about the installation of new 5G base stations during the time of the COVID-19 lockdown eventually also appear to have initiated the most visible and violent phase in the timeframe we examine here: the increase in attacks on 5G masts and towers. By 30 April, *ZDNet* counts 61 ‘suspected arson attacks’ in the United Kingdom alone, with further attacks in ‘the Netherlands, Belgium, Italy, Cyprus, and Sweden’ (Osborne, 2020).

It remains unclear whether these may have been influenced at least indirectly by the rumours about the installation of 5G in schools in the United States that had circulated since mid-March, but from 30 March onwards we see the circulation of a lengthy new post, especially in southern Africa, that not only claims that 5G technology is being installed ‘everywhere worldwide’ under cover from ‘the fake corona virus lockdown’, but explicitly states that ‘all these technologies need to be destroyed to melt . . . Fire destroys all’. Positioning perceived enemies from global elites to the freemasons, and from European royal houses to transnational corporations, as the secret actors behind the crisis, the posts includes links to a lengthy ‘5G Apocalypse’ conspiracy video on *Brighteon* (itself posted on 23 March) as well as a (now removed) YouTube video by the far-right conspiracy theorist Mark Steele (from 27 March) that discusses the installation of 5G on school buildings and describes this rollout as an ‘assault on our children’. In various versions, the post reaches some 113 spaces with at least 7.8 million followers; it has since been removed from Facebook.

Most explicitly calling for arson attacks on 5G equipment, it is possible that this post served as the point of ignition for the wave of such attacks that occurred especially in the United Kingdom in the following days. Although the circulation of the initial post centred especially on southern Africa, we note that a number of Facebook spaces with nearly identical names (‘STOP 5G & SMART METERS’, combined with the names of northern UK cities such as Dundee, Edinburgh, Leeds and Manchester) also shared this post. Although small themselves (with fewer than 500 followers in each case), they may have served as an entry point for this more violent aggression against 5G technology into the United Kingdom.

A lengthy video, published on YouTube on 2 April by the notorious conspiracy theorist David Icke, claims that COVID-19 itself is a scam and is instead caused by 5G technology. Eventually taken down by YouTube, but also republished through other video sharing sites, the video reached some 224 spaces with at least 2.8 million followers; Icke also released a further video, repeating claims that COVID-19 itself did not exist and that 5G was instead preventing human bodies from absorbing oxygen, thus causing COVID-like symptoms. Posted on the video sharing site Vimeo on 7 April, the video reached some 229 spaces with at least 4.3 million followers before it was taken down.

The conspiracy theory takes an even stranger turn with a post and accompanying videos that purport to present revelations by ‘a former executive of Vodafone’ in the United Kingdom, outlining the supposed dangers of the technology that Vodafone – ‘in partnership with Huawei’ – is now installing in the United Kingdom. Posted in a number of versions since 2 April, in English as well as in translations into Italian, Romanian and other languages, and with or without one of a range of accompanying videos, these posts were eventually shared across some 175 spaces with at least 18 million followers by the end of our timeframe. A *Guardian* investigation subsequently revealed that the person posing as a former Vodafone executive in the post and videos is in fact a Luton-based evangelical pastor from Zimbabwe, Jonathon James (Waterston, 2020).

Indeed, contributions with an apocalyptic Christian theme began to circulate widely during this phase. A post claiming that 5G technology would soon require a microchip implant, that this was intended as a form of population control in the name of the Illuminati and their god Baal, and that the election of Donald Trump and his dismantling of Obamacare had forced the shadowy group around Bill Gates that was behind this plan behind this to shift its implementation from the United States to the ‘ungodly nation’ of China, with support from Huawei, emerged first in French language among French-African communities and in Francophone Africa itself. Posted on 29 March, this version of the story reached some 253 spaces with at least 28.8 million followers.

From 4 March onwards, we also see major impact from the viral dissemination of a sermon by the Nigerian Pastor Chris Oyakhilome, who similarly claims that COVID-19 and 5G are being used to bring about a mass vaccination that aims to control the population; it is possible that his English-language sermon was influenced by the earlier French text. These claims were widely disseminated in a growing number of versions especially across Facebook spaces in central and southern Africa, to the point where it became impossible for us to reliably identify all of the various text, image meme and video versions of the sermon; however, even the 10 most widely shared versions alone appeared on 397 spaces with a combined total of at least 43.6 million followers. Such dissemination would have very substantially amplified the COVID/5G conspiracy theory particularly among African Facebook users, but also reached evangelist Christian communities well beyond that continent, from India through South Korea to Papua New Guinea.

A further version of the earlier French text, now in English and replacing substituting the freemasons for the Illuminati as the shadowy group behind these plans, had circulated across 464 spaces with more than 80 million followers by the end of our data gathering timeframe on 12 April. Here, too, circulation was especially widespread in Anglophone African Facebook spaces, and some versions of the post also directly referenced the earlier sermon by Pastor Chris Oyakhilome.

Again, this phase also sees further endorsement and amplification of COVID/5G conspiracy theories by celebrities. On 1 April, actor Woody Harrelson posts a video on Instagram that purports to show an attack on a 5G tower, yet what is in fact depicted in the video is an attack on a surveillance mast during the civil rights protests in Hong Kong (Evon, 2020); Harrelson deleted his post when this error was pointed out to him. As with the earlier Keri Hilson case, the media stories covering Harrelson’s post are mainly critical, but nonetheless circulate COVID/5G concerns to a new audience; posts from the entertainment sites *Too Fab* and *Being* alone reached at least 15 million followers through their spaces.

On the same day, UK boxer Amir Khan posted a video on Facebook (now removed) and YouTube (still available) that disputes the link between the illness and coronavirus and instead suggests that 5G technology is responsible and was being used for population control. Although the video gained relatively limited circulation in its own right, an article from the sports news site *Give Me Sport* covered these claims and embedded the full YouTube video, circulating it to the at least 25.7 million followers of its Facebook space on 5 April.

On 4 April, the US rapper Wiz Khalifa posts the simple status message ‘Corona? 5g? Or both?’ that serves as the title for this article to his group of more than 39.5 million followers. Although the post contains no further links or other information, this in itself may have led some of these followers to google for these terms and discover the by now readily available conspiracist material seeking to draw a link between COVID-19 and 5G technology.

### The other side: fact-checks and pro-5G stories

Scientific responses to the circulation of COVID/5G conspiracy content were reported widely from early April onwards. A *BBC News* article from 5 April, discussing such responses, was posted to some 158 spaces with at least 11 million followers; another reached some 51 million followers through the *BBC News* Facebook page alone. News about the take-down of prominent conspiracist content also circulated widely: a *BBC News* article from 7 April, covering the take-down of a David Icke video from YouTube, was shared on some 189 spaces with at least 5.5 million followers, with various supportive or critical comments. By contrast, fact-checking information from the WHO appeared prominently only relatively late in the timeframe we examine here. An image post containing fact-check material, released on 10 April 2020, reached some 525 spaces with a combined total of at least 23.3 million followers.

Not all mainstream news outlets contributed so successfully to fact-checking the COVID/5G misinformation, however: for example, on 7 April even the venerable Italian broadsheet *La Stampa* published an opinion piece that actively promoted conspiracy theories about the health dangers of 5G. Removed from the newspaper site some time after publication, the article continued to circulate in a version captured by the Internet Archive, and had reached some 210 spaces with at least 3.3 million followers by the end of our collection period on 12 April.

It should also be noted that by the end of this phase, social media platforms themselves begin to take more visible action against the dissemination of such conspiracy theories. Facebook, in particular, commits itself to ‘taking aggressive steps to stop misinformation and harmful content from spreading’ (Quinn, 2020) – but although we do see more evidence of content takedowns in subsequent phases, such efforts fail to fully quell the spread of these conspiracy theories or to prevent them from having significant consequences in the offline world. Indeed, some recent studies show that content takedowns, fact-checks and other attempts to slow the spread of mis- and disinformation may only entrench the beliefs of committed conspiracy theorists, as – in their worldview – they provide proof that platform operators, mainstream media, state agencies and other ‘deep state’ operatives really are attempting to silence them (cf. Krämer, 2017).

Finally, the search terms used to establish our dataset also captured a considerable number of COVID-related posts that promoted the benefits of 5G technology. Seen since early February, these predominantly originate from Chinese state media outlets such as the Xinhua news agency, the newspaper sites *China Daily* and *People’s Daily* or the CGTN and CCTV television networks and cover European languages from English through Spanish to Romanian as well as Southeast Asian languages from Cambodian through Indonesian to Vietnamese. Their dissemination is largely limited to the official Facebook spaces of these outlets, whose followers generally number between 50 and 100 million.

Such stories – which might be classified as state-sanctioned counter-propaganda – cover the use of 5G technology for telemedicine in Chinese hospitals, report on the deployment of 5G-controlled robots and drones to support doctors and patients and control the lockdown, or reaffirm the country’s commitment to the rollout of 5G technology in spite of the economic downturn caused by the COVID-19 crisis. Our focus on Facebook and our search terms in Latin characters for this study necessarily mean that we are unlikely to capture any Chinese media content designed for domestic consumption in China – but from the data we do have it is clear that Chinese state media did embark on a concerted effort to present the benefits of 5G to a world audience, not least in countries where 5G conspiracy theories circulated widely.

### Network structure

**Figure 2.**
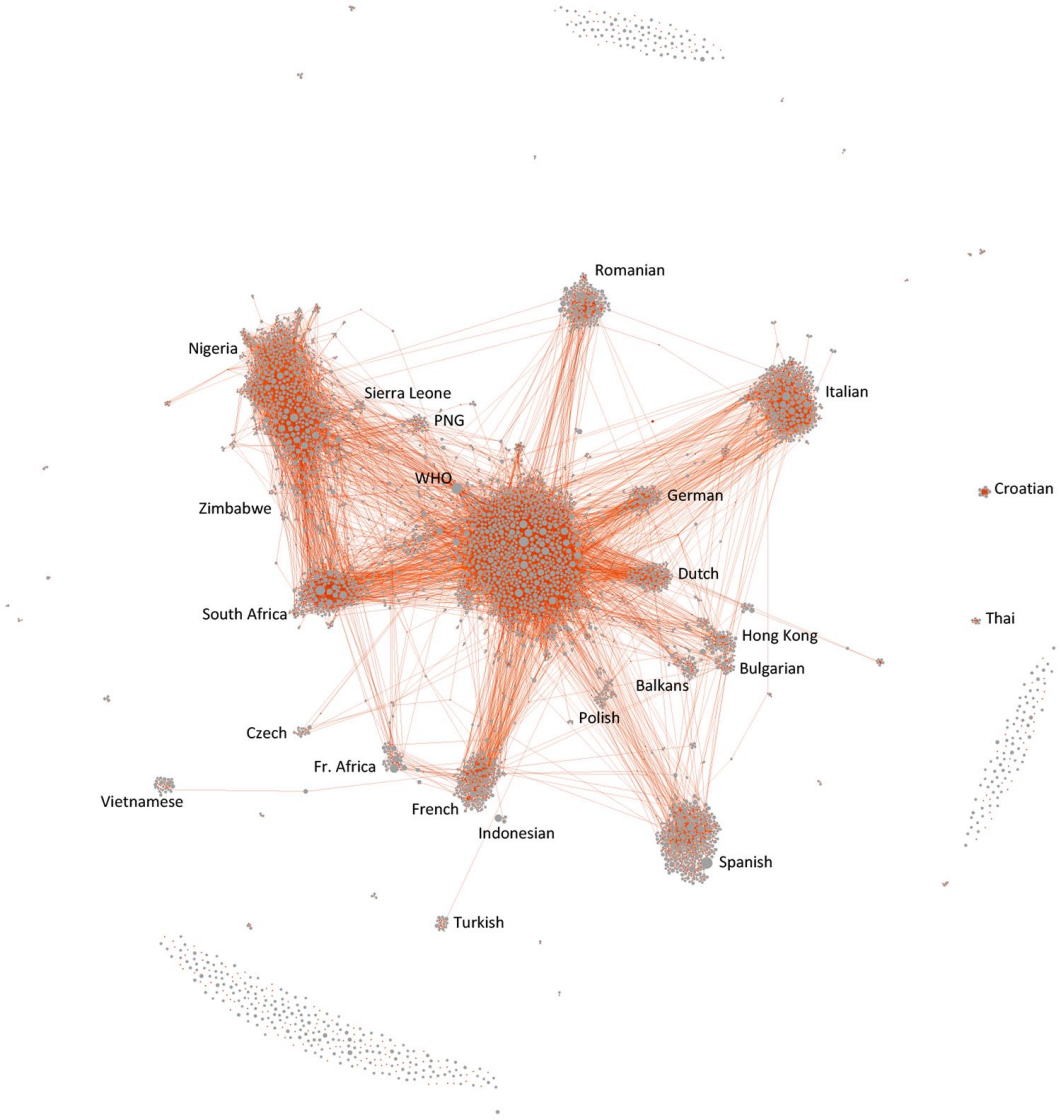
Network between Facebook spaces (red) and URLs shared (grey), filtered to a degree ⩾5.

We close this analysis with an overview of the network structure between the Facebook spaces and the COVID/5G content they shared during our timeframe. This provides additional information on the broader groupings of public spaces that are revealed by their propensity to share similar content. Figure 2 shows this as a network between the Facebook spaces (in red; as before, this includes public pages, public groups and verified profiles) and the URLs they shared (in grey; this includes both external URLs and Facebook posts shared on in other posts), visualised by the Force Atlas 2 algorithm (Jacomy et al., 2014) as implemented in the network analysis software Gephi (Bastian et al., 2009). For this visualisation, we have filtered the network to leave only Facebook spaces that shared at least five of the URLs in our dataset, and to leave only URLs that were shared at least five times. This leaves a network of 6403 spaces and URLs (11% of the total dataset), connected by 21,017 posts sharing URLs (27% of the total dataset). We have removed any Facebook spaces that did not share at least five URLs, and any URLs that were not shared at least five times, in order to reduce the network to its most active components; however, the overall structure of the network is not significantly different if all spaces and URLs are retained.

Complementing the time-series analysis that is at the core of this article, this structural evaluation of the network confirms several of our earlier observations and adds further insight. It points clearly to the presence of a number of distinct language communities that centre predominantly around the sharing of content in their own languages – these include large clusters in Italian, Spanish and French as well as (somewhat more unexpectedly) in Romanian, as well as smaller clusters in German, Dutch, Polish, Czech and interrelated Balkans languages (including Serbian, Croatian, Bosnian and Slovenian); several Asian languages are also present but generally more separate from the core of the network. Furthermore, there are a number of national and transnational African network clusters: a smaller francophone African cluster in close vicinity to the general French-language cluster, as well as larger clusters of South African and Nigerian Facebook spaces (and smaller adjacent clusters from Zimbabwe, Sierra Leone and other nations). These combine Facebook spaces that post in English as well as in a variety of local languages, and often in both.

Especially the cluster centring on Nigerian Facebook spaces is only partly determined by a shared nationality, however; there is also a strong evangelical Christian theme here, and this may also explain the location of a smaller Papua New Guinean cluster (itself containing various Christian-flavoured spaces) in its close vicinity. The relative size and interconnectivity of this African subnetwork demonstrates the significant role that African evangelical spaces have played in disseminating and popularising the COVID/5G conspiracy theory on Facebook.

Finally, at the centre of the network and serving as a connecting hub between many of these national and linguistic communities is a large and dense network of Anglophone spaces sharing COVID/5G URLs. This is thoroughly interconnected as a result of the intense cross-fertilisation between these spaces and their content; however, a close reading of the network structure does reveal further structuration: this cluster contains subsections that specialise particularly in far-right ideology (as promoted by *InfoWars* and similar sites); alternative health and spirituality; or concerns about ‘electrosmog’ radiation. This again demonstrates the unusual and disparate coalition of groups that have been brought together by conspiracy theories about the links between COVID-19 and 5G technology.

## Discussion and conclusion

At this stage, this analysis of the COVID/5G conspiracy theories and their dissemination across Facebook must necessarily remain general and descriptive; our first aim in this article has been to document in detail the trajectory of these stories from fringe circulation to significant impact over the course of little more than four months, in order to uncover the major drivers and inflection points of this gradual amplification of such outlandish and repeatedly discredited rumours and myths.

Substantial further work is now required to examine aspects such as: the role of other social media platforms in promoting these conspiracy theories, and their interconnections with Facebook itself either as staging areas for conspiracists before circulating these rumours more broadly or as follow-on channels for subsequent circulation; the degree to which what Facebook describes as ‘coordinated inauthentic behaviour’ (Gleicher, 2018), including automated posting across a number of spaces at the same time, played a role in achieving widespread dissemination of these stories; the impact of content warnings and takedowns for Facebook posts and videos, as well as for external materials such as YouTube videos, on the subsequent sharing of such content; the overlap between the Facebook spaces reached by conspiracist material and those that saw the circulation of mainstream news and fact-checking content; or the potential cyclical amplification of conspiracy material as a result of such mainstream coverage, no matter how well-intended (Phillips, 2018). We intend to conduct some of this work ourselves, and encourage other colleagues to conduct similar analyses on this and other conspiracy theories.

It is already evident from our present analysis, however, that the COVID/5G conspiracy theory is a product of the collision of long-standing conspiracist beliefs about the supposed health dangers of 5G, as well as about vaccines, global elites, China and other well-established targets of suspicion, with the emerging coronavirus crisis. Conspiracy theorists on Facebook both swiftly retro-fitted the new information emerging about the virus, its suspected origins, its effects on human health, and its likely remedies into their pre-existing worldviews, beliefs, and ideologies, and cynically exploited the genuine fears of their fellow Facebook users about the impacts this virus would have on themselves and their loved ones in order to promote their conspiracist narratives and prompt the further on-sharing of their counter-factual messages. Such processes are unlikely to be limited to Facebook alone, however; they will have unfolded in similar ways, but adjusted to the specific socio-technical affordances, on a variety of other leading social media platforms as well.

More unique to Facebook, however, is the range of communities affected by this circulation of conspiracist content. The platform’s broadly global nature (with the notable exception of countries such as China, where it remains banned; Russia, where VKontakte remains the market leader; and some other regions) enables the rapid dissemination of such content around the world, with English serving as a *lingua franca* and local and regional language communities developing their own spins on these conspiracy theories; the strong presence of specific strong-tie communities (from small but networked neighbourhood groups in many cities through communities of interest around health, spirituality or politics to large and transnational communities such as that of evangelical Christian groups especially in southern Africa) means that such communities can serve as fertile grounds for the fomentation and amplification of conspiracy theories. This may eventually also result in the circulation of key conspiracist materials (such as the major articles and videos we have highlighted in our analysis) within thematically highly divergent community spaces on the platform.

Mainstream, reliable, fact-checked information that debunks these conspiracy theories does also circulate, but less consistently and in other networks than the original conspiracist content. Such a finding is consistent with both ‘confirmation bias’ – where people generally seek out information that confirms their existing views, rather than that which challenges them (see Oswald and Grosjean, 2004) – and of social judgement theory, which posits that people are more likely to reject ideas which do not fall within a range of acceptable beliefs; sharply contradictory information is rejected, perhaps especially when the information might reject beliefs which have become central to an online identity. Indeed, even well-meaning corrections can have the opposite effect (see Nyhan and Reifler, 2010). Our data do not provide details on the specific dates at which major conspiracist content was overlaid with fact-check notices or banned from Facebook and other social media platforms altogether, and further forensic analysis will therefore be required to pinpoint the exact impact that such measures have on the further circulation of such materials; however, at least anecdotally, we can report that videos that were banned from Facebook and YouTube were re-shared in the form of new uploads to alternative platforms such as Vimeo and BitLocker and that URLs that were banned from Facebook were reposted in a deliberately misspelt form that could be easily corrected by a determined user.

Furthermore, as already noted, content removals and bans may also have the unintended effect of strengthening the beliefs of conspiracy theorists for whom such interventions are proof that they are in the process of uncovering deeper secrets that ‘the establishment’ does not want them to see. Fact-checking articles from mainstream news may also draw more attention from ordinary users to the conspiracies being debunked, and questions remain as to whether and in what instances journalists should remain ‘strategically silent’ (Benkelman, 2019). This is not in itself an argument against regulatory or mainstream media interventions, of course: they may well play a crucial role in preventing ordinary users from encountering egregiously misleading conspiracist content, but they are highly unlikely to convince an entrenched conspiracist about the errors in their worldview.

The same is likely to be true for the media coverage of celebrity endorsements. Even though much of this coverage has been immediately critical and even scathing of the musicians, actors and sports stars promoting such conspiracy content, it nonetheless also presents their statements in detail and sometimes even directly embeds their own videos or the conspiracist sources they draw on; this makes such material more immediately accessible to a large new audience that would not otherwise have encountered it. Furthermore, subsequent to such coverage we also see in our data new conspiracist posts that reference such celebrity endorsements, and even the critical media stories themselves, as new proof that the conspiracy theories are correct: from their perspective, the critical mainstream reaction to their views only indicates that they are close to uncovering some deeper truth. In the case of celebrities, there is also a concerning possibility that a combination of mainstream reaction and a newfound conspiracist audience will lead to further radicalisation. Researchers have already documented this process among popular YouTubers, following similar public controversies (Lewis, 2018). In Australia, after their outing as COVID-19 conspiracists, celebrities such as chef Pete Evans and former reality show contestant Fanos Panayides have since become highly visible spreaders of the conspiracy; Evans also sought to profit from it by selling dubious anti-COVID devices, while Panayides became a key organiser of anti-lockdown activism. Pete Evans’s recent transformation into a vocal Trump supporter, as well as his promotion of conspiracies associated with the online far right, indicates the added danger of a highly politicised radicalisation, beyond otherwise ‘milder’ social media posts that point to the (baseless) health risks of 5G. The potential for organised manipulators to target these celebrities to radicalise them further is an additional issue here.

It would be inappropriate for us to outline a comprehensive range of other, more effective countermeasures to mis- and disinformation here; many more in-depth case studies of the dissemination processes for widespread conspiracy theories, similar to ours, are needed to better understand the typical patterns and processes by which such ideas progress from fringe beliefs to mainstream impact before we arrive at a clearer picture of the key points at which their dissemination may be slowed or halted. We hope, however, that the present analysis contributes substantially to that aim by tracing in detail the developments around the nonsensical belief that 5G technology could cause or exacerbate the symptoms of a severe viral infection.

## Notes

1. Similarly, it has been argued that the myriad conspiracies regarding the terror attacks of 11 September 2001 are so enticing because the official version of events forces us to face the uncomfortable truth that such death and destruction could be caused by 19 men wielding only box-cutters.
